# Dry cupping therapy combined with conventional therapy does not provide additional benefits over conventional therapy alone in patients with non-specific chronic low back pain: a randomized trial

**DOI:** 10.1186/s12998-025-00588-x

**Published:** 2025-06-16

**Authors:** Renjie Xu, Yun Yang, Chengjie Yan, Zhou Li, Chaochen Zhao, Jingming Ma, Guangxu Xu

**Affiliations:** 1 Rehabilitation Treatment Department, Kunshan Rehabilitation Hospital, Kunshan, Jiangsu China; 2 School of Rehabilitation Medicine, Nanjing Medical University, Nanjing, Jiangsu China; 3 Department of Rehabilitation Medicine, The First Affiliated Hospital of Nanjing Medical University, 300 Guangzhou Road, Gulou District, Nanjing City, Jiangsu China; 4 Department of Neurological Rehabilitation, Kunshan Rehabilitation Hospital, Kunshan, Jiangsu China

**Keywords:** Cupping, Chronic non-specific low back pain, Pain pressure threshold, Disability, Pain

## Abstract

**Purpose:**

Chronic non-specific low back pain (CNLBP) is a complex and heterogeneous condition, and it is necessary to explore new treatment approaches. We evaluated whether the addition of dry cupping therapy to guideline‑based conventional therapy would further improve clinical outcomes in CNLBP.

**Methods:**

Thirty-six patients with CNLBP were recruitedand randomly divided into two groups: the control group and the intervention group. The intervention group received cupping therapy in addition to the control group (core stabilization exercises, spinal manipulation and education) for 4 weeks. The primary outcome was the visual analog scale (VAS) for pain intensity. Secondary outcomes were the Roland Morris disability questionnaire (RMDQ), and pressure pain thresholds (PPT) at bilateral Shenshu (BL23), Qihaishu (BL24), and Dachangshu (BL25) acupuncture points.

**Results:**

At week 4 the between‑group difference in resting pain was trivial (median difference 0.0 cm, 95% CI − 1.0 to 1.0). Neither clinically important nor statistically significant differences were detected in disability or PPTs. Both groups improved substantially from baseline.

**Conclusion:**

In this randomized trial, adding dry cupping to conventional therapy offered no additional benefit over conventional therapy alone for pain, disability or PPT in CNLBP. Larger, multicentre trials with longer follow‑up and standardized negative pressures are warranted.

*Trial registration*: ChiCTR2300069398, http://www.chictr.org.cn, Registration Date: March 15, 2023.

**Supplementary Information:**

The online version contains supplementary material available at 10.1186/s12998-025-00588-x.

## Introduction

Chronic non-specific low back pain (CNLBP), defined as persistent lumbosacral discomfort lasting ≥ 12 weeks without identifiable pathological etiology, constitutes the predominant subtype of chronic low back pain (CLBP) [1]. This multifactorial condition, accounting for 80%-90% of CLBP cases globally, has emerged as the primary cause of disability among musculoskeletal disorders globally [2, 3]. Characterized by the absence of specific pathoanatomical correlates (e.g., infection, neoplasm, spinal fracture, or inflammatory arthropathy), CNLBP predominantly involves functional musculoskeletal dysfunction rather than structural pathology [4].

One study suggested that microdamage or fatigue in the lower back due to mechanical load during daily activities or work is a significant contributing factor to CNLBP [5]. Additionally, factors such as smoking, obesity, depression, and aging also increase the risk of CNLBP [2, 6].

The management of CNLBP is highly individualized, as not all patients respond uniformly to the same interventions [7]. No single treatment has been found to be universally effective, necessitating the use of evidence-based, multifaceted rehabilitation strategies to alleviate pain and reduce treatment costs [7]. In light of the limited efficacy and potential side effects of pharmacological treatments, non-pharmacological approaches such as physical rehabilitation, health education, and complementary therapies have gained prominence in the management of CNLBP [8, 9]. Core stabilization exercises enhance spinal stability and motor control [10], while spinal manipulation therapy reduces pain sensitivity through sensory and motor modulation [11, 12]. Although both are common, their variable outcomes highlight the need for complementary therapies to address diverse patient needs [13, 14].

Cupping therapy is a traditional Chinese treatment with a history of over 4000 years, including dry cupping therapy (utilizing controlled suction without epidermal disruption) and wet cupping therapy (combining vacuum application with subsequent therapeutic phlebotomy), which has emerged as a promising complementary treatment for CNLBP [15, 16]. Cupping therapy involves creating negative pressure on the skin using vacuum-sealed cups, and it has been widely utilized to alleviate pain and improve physical function [17]. The mechanisms by which cupping therapy exerts its effects are multifaceted and include both physiological and psychological pathways. From a physiological perspective, cupping therapy improves local blood circulation, promotes the removal of pain-inducing substances, and enhances the delivery of nutrients to the affected tissues [18]. It also stimulates mechanoreceptors and small-diameter nerves, leading to the release of endorphins and other neurotransmitters that block pain signals [19]. Moreover, the mechanical deformation caused by negative pressure can restore lumbar fascia sensory feedback, improving muscle coordination and spinal stability [20]. Beyond its physiological benefits, cupping therapy often provides psychological relaxation, which may contribute to its therapeutic effects [21, 22].

Despite its widespread use and reported safety, the efficacy of cupping therapy for CNLBP remains controversial due to a lack of high-quality evidence [13]. Previous research, such as studies examining the impact of cupping therapy on muscle stiffness in the triceps, has demonstrated its potential to modulate tissue properties and reduce stiffness [23]. These findings suggest that similar benefits may extend to other regions, including the lower back. Building on this foundation, our study aimed to investigate the short-term effects of adding cupping therapy to conventional rehabilitation in patients with CNLBP. Our hypothesis was that patients who received cupping therapy in addition to conventional therapy would experience improvements in pain, physical function, and pressure pain threshold (PPT) compared to those who received conventional therapy alone.

## Materials and methods

### Study design

This was a randomized, comparative trial with single-blind and parallel groups. Our trial design strictly adhered to the STRICTOC (Standards for reporting interventions in clinical trials of cupping) checklist, with full methodological compliance details documented in Online Appendix 1 [24]. The researchers involved in the study, with the exception of the experimental designer and therapists, were unaware of the participants' identities. Additionally, the participants in both groups were also kept unaware of the intervention provided to the other group. The study was registered at www.chictr.org.cn (Registration Date: March 15, 2023), and was approved by the Ethics Committee of Kunshan Rehabilitation Hospital (No. 2023-LYP-001).

### Participants

Participants were recruited through posters and Wechat software. The study commenced in March 2023 and concluded in June 2023. The inclusion criteria for participants were as follows [25]: ages ranging from 18 to 48 years, chronic pain lasting for more than 3 months, pain between the costal margin and the transverse lines of the buttock, and pain scores (VAS) ranging from 3 to 8 points. The exclusion criteria included individuals who had received rehabilitation in the last three months, presence of dermatosis in the cupping area, painkillers for the last three months, history of previous spinal surgery, radiating pain, inflammatory or rheumatic diseases of the spine, severe pathological conditions of the spine, and other specific low back pain (LBP) with definitive underlying etiology. Physicians were involved throughout the recruitment process. They assessed participants and performed further diagnostic clarification and management if low back pain with red flag signs was identified.

### Randomization

The randomization process was simple, with a researcher who was not involved in the subsequent research stages mixing prepared cards containing therapeutic regimens in an opaque jar. Enrolled patients were randomly assigned by drawing cards from the jar.

### Blinding

This study was conducted with evaluator blinding. The participants were unaware of which group they had been assigned to. They were informed that there would be two different interventions but were not aware of the specific treatment measures in the other group. The evaluators who rated the variables were blinded as they did not know which intervention the participants had received, as they were not present during the individual interventions. Only the therapists were aware of the exact interventions.

### Interventions

**Fig. 1:**
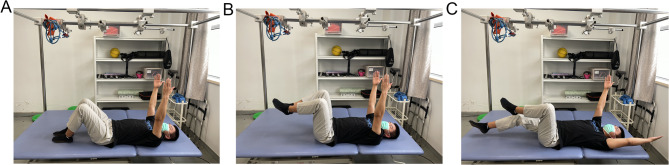
Dead bug. **A** Phase 1: Participants lay on their back with their knees bent and arms fully extended, and then alternated their arms constantly. **B** Phase 2: While performing the exercise from Phase 1, participants lifted their legs and held them. **C** Phase 3: Participants performed the dead bug exercise while alternating both their arms and legs

The participants had no prior experience with core stabilization training, spinal manipulation, or cupping therapy (Fig. 1). The spinal manipulation and partial core stabilization training were administered in the climate-controlled rehabilitation hall of Kunshan Rehabilitation Hospital, where ambient temperature was maintained at a constant 24 °C. This detailed description has been structured following the template for intervention description and replication (TIDieR) checklist to ensure replicability [26].

All participants received standardized pre-enrollment health education by a therapist, which included self-management of CNLBP, stretching, strengthening exercises, and the role of emotions and fear avoidance.

### Control group (conventional therapy only).

Participants first received spinal manipulation administered by a therapist with over 3 years of professional experience [14], followed by therapist-guided core stability training [27], as detailed in the Online Appendix 2.

### Intervention group (dry cupping + conventional therapy)

**Fig. 2:**
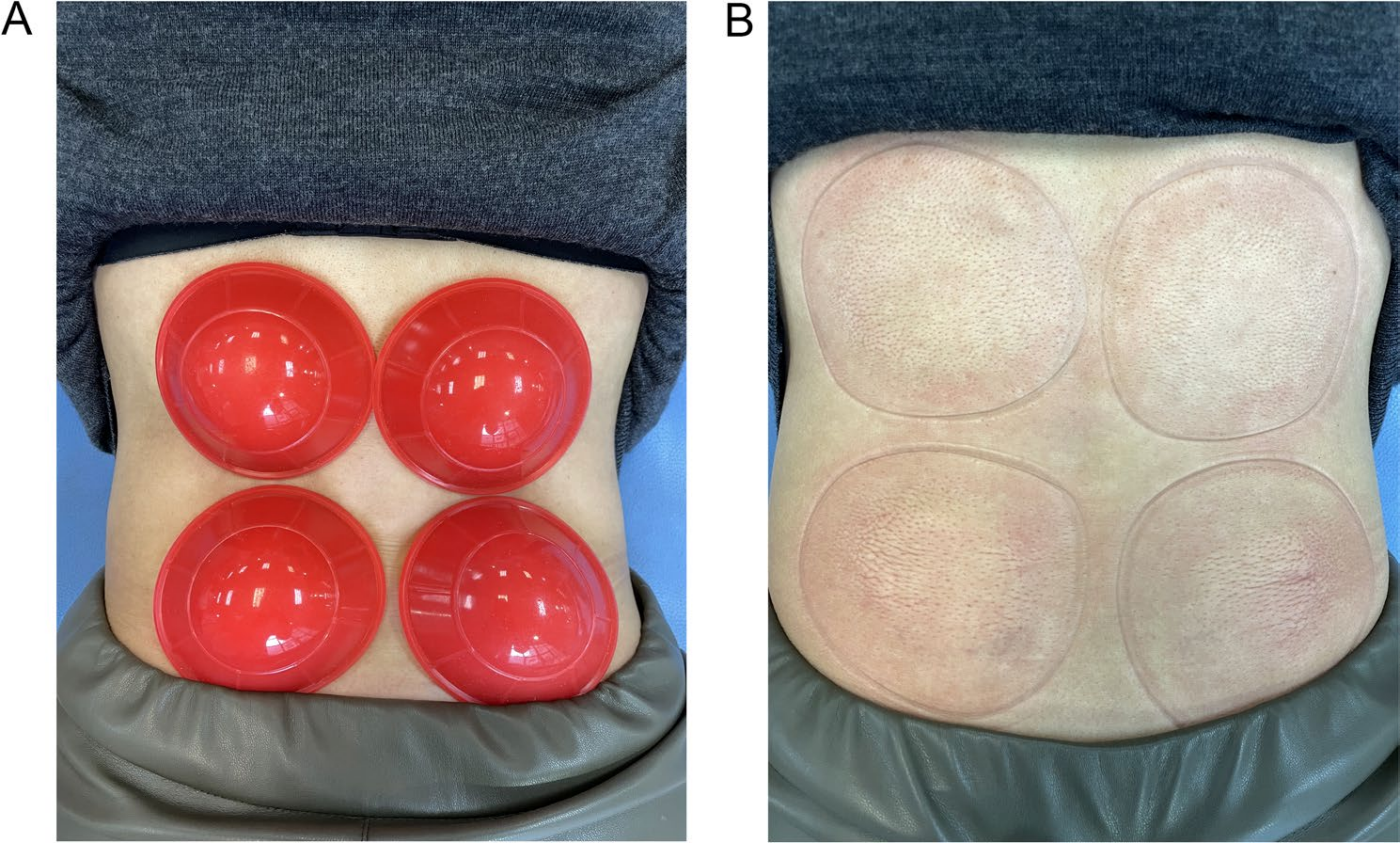
Cupping treatments. **A** Application of the silicone cups at the low back area. **B** Ten minutes after cupping

Participants in the intervention group received cupping therapy in addition to the standard treatment protocol administered to the control group. The cups used in this study are made of silicone and have an inner diameter of 5.5 cm and an outer diameter of 7.5 cm. Prior to treatment, the cups were sterilized using 70% alcohol. Subjects were positioned in a prone position on the treatment bed, allowing for the skin of the treatment area to be exposed. The therapist applied two cups in parallel on each side of the L1–L5 vertebral body and removes as much air from the cups as possible while adsorbing. Cupping therapy was performed twice a week with an interval of more than 3 days for a total of 4 weeks (Fig. 2A, 2B).

### Blinding of participants

Participants attended scheduled rehabilitation sessions at the hospital, with staggered scheduling across groups to minimize inter-group contact. However, within-group concurrent training sessions might occur during the rehabilitation process.

### Sample Size

The sample size calculation was conducted based on the results of the primary outcome, which was measured using the VAS. The sample size was determined using the G*Power 3.1 software, with a significance level (α) of 0.05 (two-sided) and a power (1-β) of 0.8, assuming an effect size of 1. The effect size of 1 was based on a similar study by Salemi et al. [28], the cupping group had a VAS mean of 2.25 with a standard deviation (SD) of 1.71, while the sham group had a mean of 4.59 (SD = 2.18). The calculated total sample size was 34 patients. To account for a potential loss rate of 15%, a total of 40 patients were enrolled in the study.

### Outcome measures

The pain intensity, disability, and PPT of both groups of subjects were assessed at baseline (T_0_) and after 4 weeks of treatment (T_4_). We conducted an additional evaluation of the PPT of both groups of subjects 10 min after the first treatment (T_1_).

### Primary outcome measures

The VAS was utilized to measure the self-reported pain intensity of the patients. The involved participants placing a marker on a 10-cm-long straight line that had stops at each end. The left stop represented no pain, while the right stop represented the worst pain imaginable [29]. MCID for VAS in LBP was 22.5 [30].

### Secondary outcome measures

The Roland Morris disability questionnaire (RMDQ) was employed to evaluate functional disability resulting from low back pain. The RMDQ is a concise and user-friendly self-assessment tool designed to measure physical function in individuals with back pain. Its simplicity makes it suitable for monitoring the progress of individual patients in clinical settings. The questionnaire comprises 24 questions that specifically address routine activities of daily living. Each affirmative response is assigned 1 point, and the final score is determined by the total number of points, ranging from 0 to 24. Higher scores indicate greater levels of disability [31]. MCID for RMDQ was 3.5 [32].

The PPT was assessed using a pressure algometer (WD, WX-100, Wenzhou, China). A pressure algometer is a device that measures the force required to elicit a PPT. These devices have been shown to have high reliability and validity [33], and they also demonstrate acceptable intraexaminer reliability in terms of pressure rate application. The PPT measurements taken across multiple sessions showed reliable results without any significant differences [34]. Refer to the study by Volpato et al. [35], PPT was measured bilaterally at three specific points: BL23 (Shenshu), BL24 (Qihaishu), and BL25 (Dachangshu). Participants were instructed to say “yes” when they started feeling pain or discomfort. Once they said “yes,” the pressure was immediately stopped, and the meter was removed from the skin. The PPT threshold was evaluated three times and averaged.

### Statistical analysis

The statistician remained blinded to group allocation throughout the data analysis process. Our study employed per-protocol analysis, with two participant withdrawals occurring in both the control and intervention groups. All remaining participants demonstrated full adherence to treatment protocols and completed all scheduled assessments, yielding complete datasets with no missing values.

Data analysis was performed using SPSS 22.0. The normality of continuous data was assessed using the Kolmogorov–Smirnov test. Normally distributed data were presented as mean ± standard deviation, while non-normally distributed data were presented as quartiles. Inter-group comparisons were conducted using the independent sample t-test, while intra-group comparisons were analyzed using the paired sample t-test. For non-normally distributed data, the Mann–Whitney test was employed. The Chi-square test was used to compare proportions in terms of sex. Significance was set at α = 0.05.

## Results

### Participant flow

**Fig. 3:**
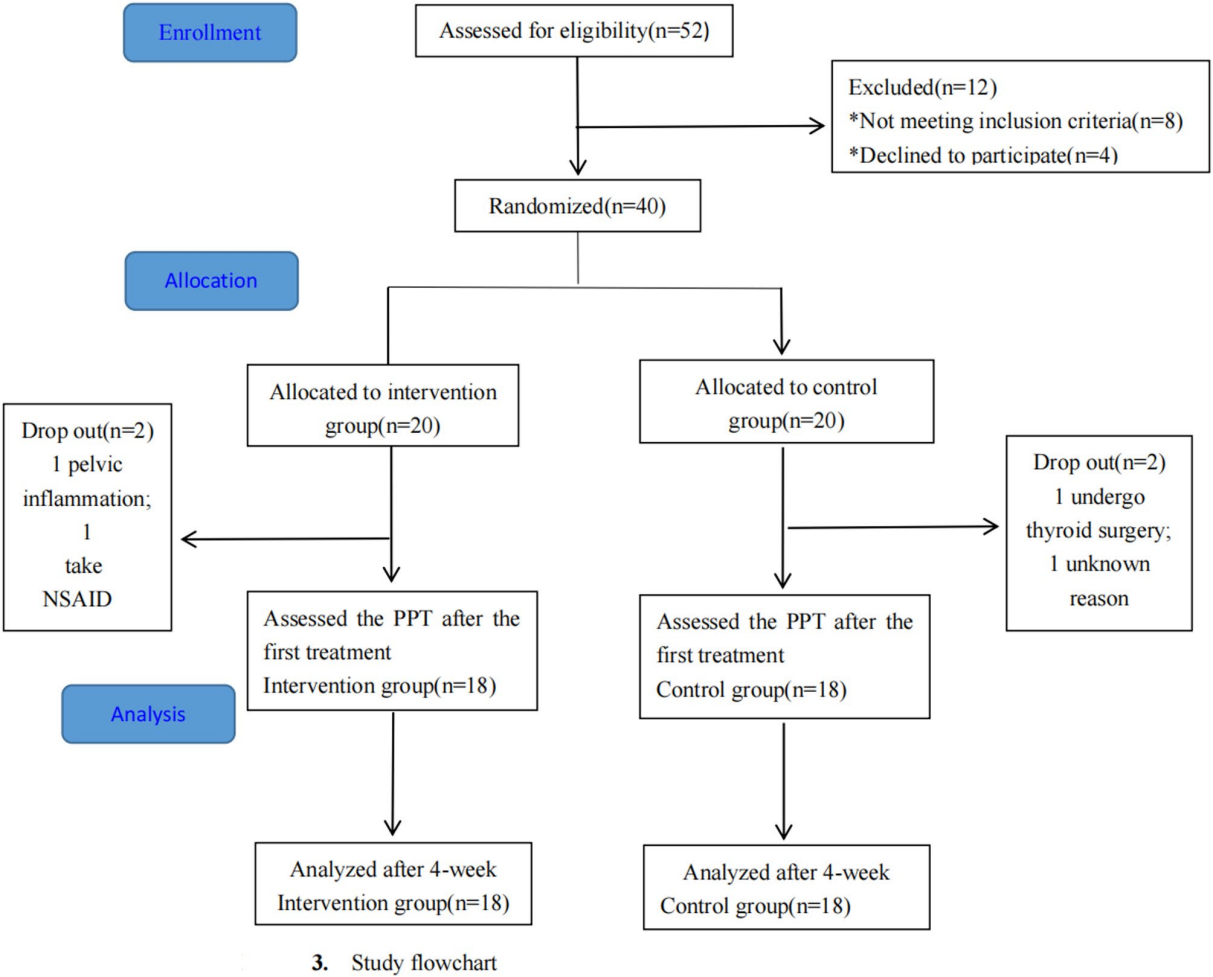
The flowchart of the study

Patients were recruited at Kunshan Rehabilitation Hospital from March 2023 to June 2023, and the study intervention and follow-up assessments were scheduled to be completed by July 2023. The recruitment and assignment process are depicted in Fig. 3.

A total of 52 eligible patients were screened, with 12 patients being excluded for not meeting the inclusion criteria (n = 8) or refusing to participate after being informed of the trial (n = 4). Finally, 40 patients were randomly assigned to either the control group or the intervention group. In the control group, one patient withdrew due to thyroidectomy, while the other did not provide a specific reason. In the intervention group, withdrawal occurred due to the sudden diagnosis of pelvic inflammatory disease and the use of nonsteroidal anti-inflammatory drugs for low back pain. Thirty‑six participants therefore completed the 4‑week protocol and were included in the per‑protocol analysis (Intervention n = 18; Control n = 18). Two intervention participants reported experiencing skin itchiness during cupping, but this sensation disappeared after the cups were removed. This acceptable adverse response persisted for two weeks. No other adverse events were documented.

Thirty-six patients with CNLBP, ranging in age from 18 to 48 years, including 6 males and 30 females, volunteered to participate in this study. The distribution of sex, age, height, weight, BMI, and duration of pain was similar between the two groups. The participants’ baseline demographic characteristics are provided in Table 1.

**Table 1:** Baseline demographic and clinical variables

	Intervention group (n = 18)	Control group (n = 18)	*P*
Age (years)^b^	32.11 (7.05)	35.83 (6.24)	0.103
Sex (male/female)^a^	4/14	2/16	0.658
Height (cm)^b^	164.50 (6.01)	163.28 (6.95)	0.576
Weight (kg)^b^	62.72 (11.45)	57.11 (9.31)	0.116
BMI (kg/m^2^)^b^	23.07 (3.19)	21.31 (2.37)	0.070
Pain duration (years)^b^	5.50 (3.76)	5.61 (3.78)	0.930
VAS^c^	5.00 (3.75, 7.25)	4.00 (2.75, 6.25)	0.323
RMDQ^c^	5.00 (3.75, 7.00)	4.00 (3.00, 5.25)	0.214

BMI: body mass index. Data are presented as mean (SD), number of patients, and median (Q1, Q3); ^a^ Chi-square test (Fisher, 2side), ^b^Independent samples *t* test. ^c^Mann-Whitney U test

### Effect of the intervention

#### Primary outcome

##### VAS

As shown in Table 2, a comparison of the VAS scores between the groups revealed no difference after 4 weeks of treatment (MD 0.00 95% CI − 1.00 to 1.00). However, compared to the baseline scores [control group: 4.00 (2.75, 6.25), intervention group: 5.00 (3.75, 7.25)], both groups experienced a significant reduction in VAS scores after 4 weeks of treatment [control group: 1.50 (0.75, 3.00), intervention group: 1.00 (0.00, 3.00)] (95% CI − 4.00 to − 1.50 and − 5.00 to − 2.00).

**Table 2:** Pain intensity and disability

	Intervention group(n = 18)	Control group(n = 18)	CI 95%	Median difference intervention/control	CI 95% (Median difference)
VAS					
T0	5.00 (3.75, 7.25)	4.00 (2.75, 6.25)	− 1.00 to 2.00	0.00( − 1.25, 4.00)	− 0.91 to 2.46
T4	1.00 (0.00, 3.00)	1.50 (0.75, 3.00)	− 1.00 to 1.00	0.00( − 1.00, 0.50)	− 1.34 to 1.12
CI 95%	− 5.00 to − 2.00	− 4.00 to − 1.50			
RMDQ					
T0	5.00 (3.75, 7.00)	4.00 (3.00, 5.25)	− 1.00 to 2.00	0.00( − 1.25, 3.00)	− 0.60 to 2.16
T4	1.00 (0.00, 3.25)	1.00 (0.00, 2.00)	− 1.00 to 1.00	0.00( − 1.00, 1.00)	− 0.50 to 1.61
CI 95%	− 4.50 to − 2.50	− 4.00 to − 2.50			

*VAS* Visual analog scale; *RMDQ* Roland Morris disability questionnaire. T_0_: Baseline; T_4_: at 4 weeks treatment. Data was presented as median (interquartile range). Mann–Whitney U test, Hodges-Lehmann 95% CI

#### Secondary outcomes

##### RMDQ

As shown in Table 2, a comparison of the RMDQ scores between the groups revealed no difference after 4 weeks of treatment (MD 0.00 95% CI − 1.00 to 1.00). However, the scores in each group significantly reduced from baseline [control group: 4.00 (3.00, 5.25), intervention group: 5.00 (3.75, 7.00)] to 4 weeks after treatment [control group: 1.00 (0.00, 2.00), intervention group: 1.00 (0.00, 3.25)] (95% CI − 4.00 to − 2.50 and − 4.50 to − 2.50).

##### PPT

A comparison of the PPT between the groups revealed no difference after 4-week interventions [95% CI: BL23 (L:  − 0.63 to 1.88; R:  − 0.44 to 1.99); BL24 (L:  − 0.64 to 1.86; R:  − 0.84 to 1.66); BL25 (L:  − 0.65 to 1.81; R:  − 0.65 to 1.92)]. However, compared to baseline, the PPT in the intervention group significantly increased after the 4 weeks of treatment [95% CI: BL23 (L:  − 1.68 to − 0.52; R:  − 1.48 to − 0.33); BL24 (L:  − 1.76 to − 0.48; R:  − 1.39 to − 0.18); BL25 (L:  − 1.70 to − 0.73; R:  − 1.76 to − 0.71)], while the PPT in the control group showed no statistical differences after the 4 weeks of treatment [95% CI: BL23 (L:  − 1.05 to 0.29; R:  − 0.89 to 0.25); BL24 (L: − 1.04 to 0.42; R: − 1.15 to 0.22); BL25 (L: − 1.17 to 0.52; R:  − 0.93 to 0.54)]. Additionally, after the first treatment, the PPT in the intervention group was significantly higher than before treatment [95% CI: BL23 (L: − 1.70 to − 1.13; R:  − 1.60 to − 0.72); BL24 (L: − 1.59 to − 0.96; R: − 1.65 to − 0.74); BL25 (L: − 1.94 to − 1.25; R: − 1.98 to − 1.10)], and it was also higher than that in the control group at the same time point [95% CI: BL23 (L: 0.03 to 2.26; R: 0.11 to 2.41); BL24 (L:0.01 to 2.25; R:0.15 to 2.51); BL25 (L:0.01 to 2.28; R:0.12 to 2.38)]. Please refer to Table 3 for further details.

**Table 3:** Pressure pain threshold

Group	Time	BL23		BL24		BL25	
		Left	Right	Left	Right	Left	Right
Intervention group (n = 18)	T_0_	4.93 ± 1.52	5.26 ± 1.54	4.88 ± 1.51	5.01 ± 1.42	4.42 ± 1.42	4.45 ± 1.37
	T_1_	6.34 ± 1.71^c^	6.42 ± 1.84^c^	6.15 ± 1.68^c^	6.20 ± 1.92^c^	6.02 ± 1.78^c^	5.92 ± 1.76^c^
	T_4_	6.03 ± 2.01^b^	6.16 ± 1.91^b^	5.99 ± 2.06^b^	5.79 ± 2.02^b^	5.63 ± 1.81^b^	5.69 ± 2.01^b^
Control group (n = 18)	T_0_	5.02 ± 1.49	5.07 ± 1.54	5.07 ± 1.73	4.92 ± 1.62	4.73 ± 1.57	4.86 ± 1.63
	T_1_	5.20 ± 1.57^a^	5.16 ± 1.53^a^	5.03 ± 1.62^a^	4.87 ± 1.55^a^	4.87 ± 1.56^a^	4.72 ± 1.73^a^
	T_4_	5.40 ± 1.67	5.39 ± 1.68	5.38 ± 1.61	5.38 ± 1.64	5.05 ± 1.83	5.06 ± 1.77

BL23: Shenshu; BL24: Qihaishu; BL25: Dachangshu. T_0_: baseline; T_1_: at the first treatment; T_4_: at 4 weeks treatment. Date was presented as mean (standard deviation); Independent samples *t* test and paired sample* t* test. Inter-group: ^a^*P* < 0.05; intra-group (T_4_ vs T_0_): ^b^*P* < 0.05; (T_1_ vs T_0_): ^c^*P* < 0.05.

## Discussion

This study investigated the short-term effects of adding cupping therapy to conventional rehabilitation in patients with CNLBP. Our primary objective was to compare the efficacy of conventional therapy alone versus conventional therapy supplemented with cupping therapy. Both groups exhibited significant improvements in pain intensity (VAS) and disability (RMDQ) after 4 weeks of treatment; however, no statistically significant differences were observed between the two groups.

The absence of between-group differences suggests that the addition of cupping therapy does not enhance the benefits of conventional treatment. Both groups received core stabilization exercises, spinal manipulation, and patient education, which are well-established interventions for CNLBP [36]. Our findings substantiate that the conventional therapeutic regimen integrating health education, core stabilization training, and spinal manipulation demonstrates statistically significant improvements in both pain mitigation and disability index reduction among patients with CNLBP, with clinically satisfactory short-term outcomes. These well-established integrative treatments may have minimized the additional impact of cupping therapy on short-term outcomes. Our findings are consistent with the previous study [21], which found that cupping therapy was not superior to sham interventions for improving pain, function, or quality of life in CNLBP patients. The overall effect of cupping therapy on the multifactorial nature of CNLBP appears to be limited.

Regarding secondary outcomes, the intervention group exhibited a transient improvement in PPT immediately after the first treatment session, suggesting an immediate neuromodulatory effect. The immediate PPT increase observed in the intervention group highlights the potential of cupping therapy to modulate pain sensitivity through immediate physiological effects. However, after 4 weeks of treatment, although the PPT in the intervention group remained higher than baseline, it was not significantly different from the control group. This may be attributed to the timing of the final assessment, conducted 3–5 days after the last treatment session, during which elevated PPT levels may have gradually returned to baseline, as observed by Suzuki et al. [37].

However, some studies have reported significant long-term benefits of cupping therapy for pain reduction and functional improvement. These findings diverge from our own observations. These discrepancies may be attributed to differences in study protocols and populations. For example, Volpato et al. [35] and Salemi et al. [28] only included single sessions of cupping and sham cupping in their studies, whereas we incorporated combination treatment in our trial. Additionally, cultural familiarity with cupping therapy among our Chinese participants could have influenced their response, potentially reducing the placebo effect compared to studies conducted in populations less exposed to this practice. Variability in the standardization of negative pressure applied during cupping, as well as differences in adjunctive treatments, may also explain the inconsistencies across studies.

In our study, two participants in the intervention group reported mild pruritus during moving cupping therapy sessions. These self-limiting reactions resolved spontaneously by the fourth treatment session without recurrence. This finding highlights the clinical imperative to thoroughly inform patients of potential transient cutaneous responses (e.g., erythema, pruritus) prior to treatment initiation, thereby optimizing safety profiles in manual therapy interventions.

In summary, while cupping therapy may provide immediate, short-term effects on pain sensitivity, our study demonstrates that it does not offer additional long-term benefits in pain relief or functional improvement when combined with conventional therapy.

### Strengths and limitations

This study has significant implications for clinical practice, indicating that for patients with chronic nonspecific low back pain, the conventional treatment combining health education, core stabilization exercises, and spinal manipulation demonstrates sufficient therapeutic efficacy, thereby eliminating the necessity for supplementary cupping interventions. Our study had certain limitations. First, the utilization of silicone cups, while convenient and practical, limited the ability to precisely standardize the negative pressure applied during cupping therapy, potentially contributing to variability in treatment effects. Second, the follow-up period was relatively short, focusing on immediate and short-term outcomes without assessing the long-term sustainability of the observed benefits. Third, the intervention group received cupping therapy twice weekly, while the treatment group underwent spinal manipulation once per week. This discrepancy in total intervention times may introduce confounding effects on the outcomes. Additionally, while a sham control group could have provided a stronger control for placebo effects, it was not included due to the cultural familiarity of participants with cupping therapy, which could have compromised the blinding and validity of a sham intervention. Lastly, the single‑centre design and modest sample size resulted in limited statistical power and generalisability, particularly to older adults and other care settings. Future studies should employ standardized protocols to ensure consistent treatment application, extend follow-up periods to evaluate long-term effects, recruit larger and more diverse samples to improve generalizability, and incorporate appropriate control groups or innovative placebo designs to better distinguish the specific effects of cupping therapy.

## Conclusion

The addition of dry cupping to standard rehabilitation for CNLBP did not yield greater improvements in pain or disability compared to conventional therapy alone over a four-week period. The improvements observed in both groups may reflect the general effectiveness of the core rehabilitation program rather than a distinct effect of cupping. Considering the study’s limitations, such as the modest sample size, single-center setting, short follow-up period, and absence of a sham control, further research through larger, multicenter studies with longer follow-up is needed before cupping can be considered for routine clinical use in this context.

## Supplementary Information

Supplementary material 1.

Supplementary material 2.

## Data Availability

The datasets generated during and/or analyzed during the current study are available from the corresponding author on reasonable request.
