# Supplementary material for: Dry cupping therapy combined with conventional therapy does not provide additional benefits over conventional therapy alone in patients with non-specific chronic low back pain: a randomized trial
Source: Chiropr Man Therap. 2025 Jun 16;33:26. doi: 10.1186/s12998-025-00588-x (PMC12168300; doi:10.1186/s12998-025-00588-x)
Supplement: Supplementary file 1 — Supplementary material 1. [file 12998_2025_588_MOESM1_ESM.docx]

**STRICTOC checklist**

**1 Cupping rationale**

**1a. Style of cupping (e.g., Chinese medicine, dry cupping, wet cupping, etc.)**

Our study aims to investigate the effects of adding dry cupping therapy to conventional treatment on pain, functional outcomes, and pressure pain threshold in patients with chronic non-specific low back pain. We designed a two-group trial where the experimental group received conventional rehabilitation therapy, while the control group received additional cupping therapy based on the experimental group's regimen. Forty participants with chronic non-specific low back pain were recruited from Kunshan Rehabilitation Hospital between August 2022 and June 2023, and were randomly allocated into two groups.

**1b. Reasoning for cupping provided, based on historical context, literature sources, and/or consensus methods, with references where appropriate**

In China, cupping therapy is a widely used complementary and alternative treatment, often incorporated into comprehensive management of chronic non-specific low back pain (CNLBP). However, there remains limited high-quality evidence supporting its efficacy. To address this gap, we designed the present randomized controlled trial.

**1c. Whether the cupping treatment is individualized or not**

The cupping intervention was administered uniformly to all participants following a standardized protocol without individual adaptations.

**2 Details of cupping**

**2a. Patient posture during the cupping.**

During cupping therapy administration, participants were maintained in a standardized prone position with the lumbosacral skin surface fully exposed for clinical intervention.

**2b. Devices used for cupping, such as type of cupping set, size, manufacturer, and material (e.g., herbal, needle, moxa, water) inside the cup, if any.**

The cupping procedure required no external tools, utilizing soft silicone cups (external diameter: 7.5 cm; internal diameter: 5.5 cm) manufactured by Zhuhaokang® (Medical Device Registration Certificate: Jiheng Xiebei 20170023).

**2c. Name and number of acupoints/meridians/locations (if no ofcial name) used for cupping**

Cups were applied bilaterally to the spinous processes of L1-L5 vertebrae, specifically targeting the bladder meridian acupoints BL23 (Shenshu), BL24 (Qihaishu), and BL25 (Dachangshu), with symmetrical placement of two cups per lateral chain (total 4 cups).

**2d. Number of cupping units and/or cupping time per location (mean or range where relevant)**

All four cups were maintained for a standardized duration of 8-10 minutes per application.

**2e. Procedure and technique for cupping (e.g., weak/light cupping, medium cupping, strong cupping, moving cupping, light-moving cupping, needle cupping, hot needle and moxa cupping, empty/fash cupping, bleeding/wet/full cupping, herbal cupping, water cupping and ice cupping)**

The therapist applied two cups in parallel on each side of the L1-L5 vertebral body and removes as much air from the cups as possible while adsorbing. Cupping therapy was performed twice a week with an interval of more than 3 days for a total of 4 weeks

**2f. Responses sought from participants (e.g., warm feeling, skin reddening, ring mark, etc.)**

Participants were informed that transient ecchymosis might develop at cupping sites, which typically resolves spontaneously within 2-4 days.

**2g. Precautionary measures to adverse events (e.g., skin blister, scald, or bleeding), and management, if any**

Two participants reported tolerable pruritus during cupping application, which resolved immediately upon cup removal. This transient adverse reaction resolved completely after four treatment sessions.

**3 Treatment regimen**

**Number, frequency and duration of the cupping sessions**

The control group received standardized cupping sessions conducted over a 4-week intervention period, comprising twice-weekly applications with 8-10 minutes duration per session.

**4 Other components of treatment**

**4a. Details of other interventions administered to the cupping group (e.g., acupuncture, moxibustion, massage, herbs, exercises, lifestyle advice)**

The control group additionally received structured health education and core stability training interventions (see Appendix 2 for detailed protocols), which were provided concurrently with the standardized cupping regimen.

**4b. Setting and instruction of treatment to the cupping providers and the participants**

Prior to the initial cupping session, participants were informed of potential sensory perceptions and adverse reactions through standardized protocol briefing, along with post-intervention assessment timelines at 4-week completion. The experimental procedures were conducted in the rehabilitation therapy hall of Kunshan Rehabilitation Hospital, with environmental conditions maintained at a controlled ambient temperature of 24°C (±0.5°C).

**5 Treatment provider background**

**Description of treatment provider(s) (qualifcation or professional afliation, years in cupping practice, and other relevant experience for professional)**

The cupping interventions were administered by a licensed therapist from Kunshan Rehabilitation Hospital, who received his Bachelor's degree in Rehabilitation Medicine from Nantong University and is currently pursuing a postgraduate degree at Nanjing Medical University. This practitioner has accumulated six years of clinical expertise in standardized cupping protocols.

**6 Control or comparator of cupping**

**6a. Rationale for the choice of control or comparator of cupping**

No cupping-related control interventions were implemented in the comparison group throughout the study duration.

**6b. Precise description of the control or comparator. If another form of cupping or cupping-like control is used, provide details as for Items 1 to 3 above**

Given the established cultural familiarity with cupping therapy among Chinese participants, any methodological attempts to diminish negative pressure or administer sham cupping procedures would be readily discernible to subjects, thereby compromising blinding integrity. This inherent cultural competence necessitated the exclusion of cupping-placebo controls in our comparative group design.
