# Supplementary material for: Dry cupping therapy combined with conventional therapy does not provide additional benefits over conventional therapy alone in patients with non-specific chronic low back pain: a randomized trial
Source: Chiropr Man Therap. 2025 Jun 16;33:26. doi: 10.1186/s12998-025-00588-x (PMC12168300; doi:10.1186/s12998-025-00588-x)
Supplement: Supplementary file 2 — Supplementary material 2. [file 12998_2025_588_MOESM2_ESM.docx]

**Appendix 2**

**Spinal Manipulation**

The participant was positioned in a side-lying posture, facing the therapist, with the more painful side facing upwards. The therapist then passively flexed the participant's hips and knees, causing flexion in the lumbar spine until movement was felt in the spinous process of the affected lumbar vertebrae. The therapist passively rotated the participant's torso in the opposite direction of the side they were lying on, until rotation was felt in the vertebra above the suspected lesion. The therapist then applied a rapid thrust to the shoulder (anterior to posterior force) and pelvis (posterior to anterior force), creating a rotational force couple on the hypomobile segment. If an audible pop, known as cavitation, occurred, the treatment was considered complete. If no cavitation was produced, the participant was repositioned and the manipulation was attempted again. A maximum of 2 attempts per side was allowed. If no cavitation occurred after the 4 attempts (2 per side), the treatment was considered complete. Spinal manipulation was performed once a week, for a total of 4 sessions.

**Dead Bug Exercise**

The dead bug exercises were performed as follows:

Phase 1 (Figure 1 A): Participants lay on their back with their knees bent and arms fully extended. They then alternated their arms constantly.

Phase 2 (Figure 1 B): While performing the exercise from Phase 1, participants lifted their legs and held them.

Phase 3 (Figure 1 C): Participants performed the dead bug exercise while alternating both their arms and legs.

A therapist supervised the first few sessions to make sure they completed the sessions. Subsequent training sessions were completed by the subjects at home. Participants were permitted to advance to the subsequent training phase upon self-assessed effortless completion of the current stage’s protocol. The core stabilization exercise was performed three times a day for 5 to 10 minutes each time, for a duration of 4 weeks. After completing the training, the subjects were required to upload the records of the exercise in the Wechat group.
